# Beyond Visual Acuity: Contrast Sensitivity as a Potential Neuro‐Visual Dimension of “Eye Frailty” in Mild Cognitive Impairment

**DOI:** 10.1111/ggi.70349

**Published:** 2026-01-20

**Authors:** Yi‐Ching Chu, Chao‐Chun Huang

**Affiliations:** ^1^ Department of Ophthalmology Far Eastern Memorial Hospital New Taipei City Taiwan; ^2^ Department of Physical Medicine and Rehabilitation Far Eastern Memorial Hospital New Taipei City Taiwan; ^3^ Department of Physical Therapy and Assistive Technology National Yang Ming Chiao Tung University Taipei Taiwan

To the Editor,

We read with great interest the article by Uchida et al. [1], regarding the J‐MINT study baseline data. The authors highlighted a compelling dissociation in older adults with Mild Cognitive Impairment (MCI): while clinical visual acuity (LogMAR) was largely preserved, self‐reported visual difficulties were significantly associated with physical, social, and cognitive frailty. We commend the authors for establishing “self‐reported vision status” (SRVS) as a sensitive marker for frailty, potentially superior to standard acuity charts in this specific population.

From a neuro‐ophthalmic perspective, we suggest that this discrepancy—good acuity yet poor subjective vision—is not merely a psychological phenomenon but likely reflects specific pathophysiological changes in the early stages of neurodegeneration. Standard high‐contrast visual acuity testing primarily assesses the *parvocellular pathway*, which is often resilient in early MCI [2]. Conversely, the *magnocellular pathway* and the dorsal visual stream, which process low‐contrast information, motion, and depth, are known to be selectively vulnerable to early amyloid and tau pathology [3].

Therefore, the “poor vision” reported by participants, despite their 20/20 acuity, may represent a *functional deficit in contrast sensitivity (CS)*. This “hidden” deficit explains the study's observation that poor SRVS correlates with the Fall Risk Index (FRI) [1]. Postural stability relies heavily on detecting low‐contrast edges (like carpet borders) and processing optic flow, functions governed by the dorsal stream rather than the foveal acuity measured by LogMAR [4].

We propose that Uchida et al.'s findings support a paradigm shift in defining “Eye Frailty.” We suggest conceptualizing subjective vision not just as a proxy for acuity, but as a *distinct neuro‐visual dimension of frailty*. This dimension likely captures the “cognitive cost” of processing visual information through a compromised neural network.

Moving forward, incorporating rapid contrast sensitivity assessments (e.g., Pelli‐Robson charts or frequency‐doubling technology) in geriatric research frameworks could objectively quantify this dimension. This would bridge the gap between the patient's lived experience and clinical metrics, ultimately refining fall prevention strategies for the growing MCI population.

## Funding

The authors have nothing to report.

## Disclosure

The authors have nothing to report.

## Ethics Statement

The authors have nothing to report.

## Consent

The authors have nothing to report.

## Conflicts of Interest

The authors declare no conflicts of interest.

## Data Availability

Data sharing is not applicable to this article as no new data were created or analyzed in this study.
